# Exploring the dynamics of viscoelastic adhesion in rough line contacts

**DOI:** 10.1038/s41598-023-39932-7

**Published:** 2023-09-12

**Authors:** Luciano Afferrante, Guido Violano, Giuseppe Carbone

**Affiliations:** 1 Department of Mechanics, Mathematics and Management, Polytechnic University of Bari, Via E. Orabona, 4, 70125 Bari, Italy; 2 Department of Materials Science and Engineering, Saarland University, Campus, Geb. C6.3, 66123 Saarbrücken, Germany

**Keywords:** Mechanical engineering, Surfaces, interfaces and thin films

## Abstract

Modeling the adhesion of viscoelastic rough surfaces is a recent challenge in contact mechanics. Existing models have primarily focused on simple systems with smooth topography or single roughness scale due to the co-action of roughness and viscoelasticity leading to elastic instabilities and rate-dependent behavior, resulting in complex adhesion dynamics. In this study, we propose a numerical model based on a finite element methodology to investigate the adhesion between a randomly rough profile and a viscoelastic half-plane. Approach-retraction simulations are performed under controlled displacement conditions of the rough indenter. The results demonstrate that viscous effects dampen the roughness-induced instabilities in both the approach and retraction phases. Interestingly, even when viscous effects are negligible, the pull-off stress, i.e., the maximum tensile stress required to detach the surfaces, is found to depend on the stiffness modulus and maximum load reached during the approach. Furthermore, when unloading is performed from a relaxed state of the viscoelastic half-plane, both adhesion hysteresis and pull-off stress are monotonic increasing functions of the speed. Conversely, when retraction begins from an unrelaxed state of the material, the maximum pull-off stress and hysteretic loss are obtained at intermediate velocities.

## Introduction

Adhesion is the ability of two surfaces to stick together due to attractive interactions occurring at the molecular scale. These interactions generate tensile stresses that far exceed atmospheric pressure, implying that, in principle, the entire universe should exhibit stickiness^1^. However, adhesion is rarely observed at a macroscopic scale, as surface roughness reduce the area of contact between atoms, killing adhesion^2^. An exception to this is the case of soft viscoelastic materials, which can display macroscopic adhesion^3^.

Viscoelastic materials undergo time-dependent deformation, which can result in complex adhesion dynamics characterized by contact hysteresis^4^. The latter is the difference between the work spent to create an adhesive bond and the one required for breaking it. Viscoelastic adhesion hysteresis arises from viscous dissipation occurring in a loading cycle.

Even when viscous effects are negligible, hysteresis can occur in the case of medium (or short) range interactions, because of jump-in and jump-off contact instabilities. Such abrupt change of the contact state entails local phenomena as well as wave propagation that dissipate the energy stored into the system. Often it is assumed that such dissipative and radiative phenomena occur on time-scales much shorter that those involved in the loading-unloading process. JKR theory, for example, predicts these contact instabilities in the case of smooth elastic spheres^5^. In presence of surface roughness, multiple instabilities occur at the asperities level, leading to a complex hysteretic behavior^6^.

In presence of a viscoelastic material, one could distinguish between the energy loss due to material viscous dissipation and the amount of dissipated energy related to elastic instability as described above. While some progress has been made in understanding the mechanisms of viscoelastic adhesion^7–10^, the complex interplay between surface roughness and viscoelasticity in presence of adhesion has not been fully investigated.

Among the first attempts to address the challenges of viscoelastic rough adhesion, Violano et al.^11^ proposed a multiasperity model to describe the rate-dependent adhesion and hysteresis occurring in the normal contact between rough PDMS substrate and a glass indenter. The model distinguished the energy loss due to material dissipation from the adhesion hysteresis due to elastic instability, and results were validated with experimental data^4^. However, basic topographies were considered in the former studies, as roughness was described by a random distribution of spherical cups with identical radius of curvature.

On a geometry characterized by a line contact with single roughness scale, Pérez-Ràfols et al.^12^ demonstrated that the effects of roughness and viscoelasticity on adhesive hysteresis are decoupled and additive when viscous dissipation is confined to the contact edges. Moreover, consistent with the findings in Ref.^13^, they observed that the contact becomes stiffer as the surface waviness is increased. A similar effect is observed when the detachment speed is enhanced, causing the material response to shift towards its glassy region. In both cases, there is a transition from short-range to long-range adhesion. The former is characteristic of compliant contacts, where adhesive interactions primarily occur within the contact area. In this case, detachment mainly occurs by crack propagation. On the other hand, long-range adhesion is typical of stiff contacts, where adhesive interactions are distributed mainly outside the contact area and detachment occurs as uniform bond breaking. The transition from short-range to long-range adhesion is governed by the Tabor parameter^14^ $\mu _{\text {T}} = R^{1/3}(\Delta \gamma /E^{*})^{2/3}/\epsilon$, where $\Delta \gamma$ represents the surface energy, and $\epsilon$ denotes the range of action of adhesive forces. Here, $E^{*}$ represents the reduced elastic modulus of the system, which is defined as $1/E^{*} = (1-\nu _{1}^{2})/E_{1} + (1-\nu _{2}^{2})/E_{2}$, being $E_{1}$ and $E_{2}$, and $\nu _{1}$ and $\nu _{2}$ the elastic moduli and Poisson ratios of the contacting bodies, respectively. In the context of rough contacts, an extension of the definition of $\mu _{\text {T}}$ is often obtained by interpreting *R* as the average radius of curvature of the rough profile^15,16^. Specifically, short-range adhesion is expected when $\mu _{\text {T}}$ $\gg$ 1, while for lower values the contact is characterized by long-range adhesion.

Recently, Müller et al.^17^ performed experimental investigations on the contact between a PDMS substrate and a cylindrical flat punch with a surface featuring a single-wavelength corrugation. They also compared experimental measurements with their Boundary Element Method (BEM) predictions, studying the coaction of adhesive elastic instabilities and viscoelasticity.

To the best of the author’s knowledge, an overall model which accounts for adhesion, viscoelasticity and random roughness is not still available in the literature. In fact, real surfaces exhibit a wide range of length scales, from nanometres to millimeters^6^. Roughness can significantly affect the contact area, contact pressure, and stress distribution in soft materials, which in turn affect the adhesion^18–20^ and friction^21–25^ behaviour.

In this article, we propose a model for the adhesive contact between viscoelastic bodies with realistic randomly rough surfaces. The model considers line contacts and allows for the study of the combined effects of surface roughness and material viscoelasticity on adhesion. Our objective is to provide a comprehensive understanding of viscoelastic adhesive rough contacts, which can have important implications for the design and optimization of adhesive materials in various industrial applications, including soft grippers^26^, coatings^27^, micro- and nanoelectromechanical systems^28,29^, and biological adhesive systems^30^.

## Results

All the details about the numerical model and the employed parameters can be found in the section Methods. To capture rate-dependency of viscoelastic adhesion, we have performed normal contact simulations at fixed displacement rate $V=d\delta /dt$ of the rough indenter. In the discussion of the results, we shall refer to the normalized speed $\hat{V}=V\tau /\epsilon$ and surface energy $\Delta \hat{\gamma }=\Delta \gamma /(E_{0}h_{\text {rms}})$. For smooth Hertzian indenters, we know that (see Ref.^7^) the contact behaviour changes significantly depending on whether unloading starts from a relaxed or unrelaxed state of the viscoelastic material. A relaxed state is reached when loading is performed under quasi-static condition ($V\sim 0$). In such case the material response is elastic with constant elastic modulus $E_{0}$. This condition, for example, has been reached during contact experiments on PDMS samples in Ref.^6^ by performing the loading phase at indentation speed of the order of few nanometers per second. Alternatively, a relaxed state can be reached by waiting a sufficiently large dwell time before unloading^4,31^, to ensure that viscous dissipation disappears.

**Figure 1:**
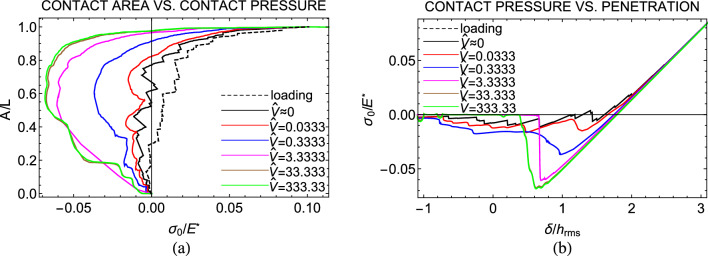
(**a**) The normalized contact area *A*/*L* as a function of the applied contact pressure $\sigma _{0}/E^{*}$ for different values of the retracting speed $\hat{V}$ and unloading starting from relaxed state; (**b**) The adimensional applied contact pressure $\sigma _{0}/E^{*}$ as a function of the normalized approach $\delta /h_{\textrm{rms}}$ for different values of the retracting speed $\hat{V}$ and unloading starting from relaxed state. Results are given for a self-affine fractal profile with Hurst exponent $H=0.8$, $h_{\textrm{rms} }=20$ nm, $\zeta =128$, and surface energy $\Delta \hat{\gamma }=0.025$.

A first set of simulations has been run with unloading starting from a relaxed state of the viscoelastic material. To this end, we have fixed a loading speed $\hat{V}\sim 0$ and performed unloading at different rates of speed.

Figure 1a shows the normalized contact area *A*/*L* as a function of the adimensional pressure $\sigma _{0}/E^{*}$, being $E^{*}=E_{0}/(1-\nu ^{2})$ the reduced elastic modulus of the system. Similarly, Fig. 1b shows the relation between $\sigma _{0}/E^{*}$ and the normalized approach $\delta /h_{\textrm{rms}}$.

The existence at the interface of a non-zero gap impedes a rigorous definition of the contact area^32,33^. For this reason, in the present context, the contact area is defined as the sum of the segments where the gap is less than a specified threshold, which is assumed to be $(g(x)-\varepsilon ) < 0.2\varepsilon$. We checked that for the case of the adhesive contact of smooth spheres, where JKR theory applies, such definition yields accurate values of the contact radius.

Both Figs. 1a and b show that adhesion hysteresis occurs in the elastic limit, i.e., when unloading is performed under quasi-static condition ($\hat{V}\sim 0$) and, hence, viscous dissipation is negligible. Carbone et al.^34^ deeply investigated such a phenomenon and proposed two different mechanisms to explain the hysteretic loss, namely the small- (SSH) and large-scale hysteresis (LSH). SSH is commonly observed in scenarios like the detachment of a parabolic indenter with wavy roughness superimposed. For this geometry, Guduru^35,36^ showed that during the unloading process, crack propagation jumps occur, leading to stable and unstable branches and resulting in increased energy dissipation at the interface. On the other hand, LSH is characteristic of a single spherical asperity, where the contact experiences a single unstable jump-off, leading to hysteretic loss. In rough contacts, the distinction between SSH and LSH is based on a threshold roughness wavelength $\lambda _{\textrm{th}}\approx \lambda _{\textrm{r}} \left[ \Delta \gamma /(E^{*}h_{\textrm{rms}})\right] ^{1/H}$, where SSH occurs for wavelengths $\lambda<$ $\lambda _{\textrm{th}}$, with each contact represented by a compact interval behaving similarly to the Guduru problem. Conversely, LSH occurs for wavelengths $\lambda>$ $\lambda _{\textrm{th}}$, where the contact at large scales consists of disconnected small contact regions resembling smooth asperities. Each of these asperities causes hysteretic loss due to local stretching and consequent JKR pull-off during the unloading process^37,38^. Notably, both SSH and LSH mechanisms can occur concurrently in the context of rough contacts and lead to an unloading path characterized by multiple instabilities.

**Figure 2:**
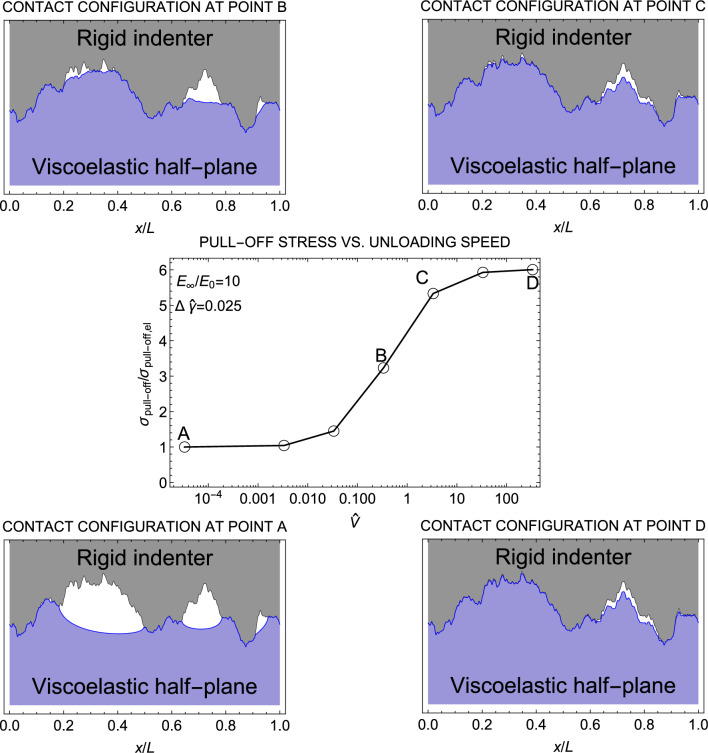
The pull-off stress $\sigma _{\mathrm {pull-off}}/\sigma _{\mathrm {pull-off,el}}$ normalized with respect to the elastic value as a function of the retracting speed $\hat{V}$ when unloading starts from relaxed state. Results are given in a semi-log plot and for a self-affine fractal profile with Hurst exponent $H=0.8$, $h_{\textrm{rms}}=20$ nm, $\zeta =128$, and surface energy $\Delta \hat{\gamma } =0.025$. In the figure, the deformed configuration of the half-plane at the pull-off instant is also shown for four unloading speeds ($\hat{V} =0, 0.3333, 3.333, 333.33$).

When unloading is instead performed at non-zero speed $\hat{V}$, viscous dissipation comes into play and adhesion can be characterized by an effective surface energy $\Delta \gamma _{\textrm{eff}}(\hat{V})$, which increases with the retraction speed, exceeding the quasi-static limit $\Delta \gamma (\hat{V}\sim 0)$^7^. Moreover, at high $\hat{V}$, the unloading path becomes smoother, as viscoelasticity reduces the effect of roughness-induced elastic instabilities^12^.

**Figure 3:**
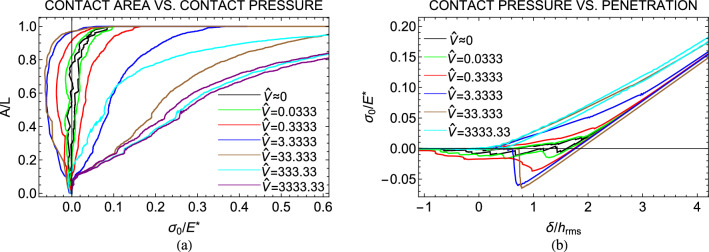
(**a**) The normalized contact area *A*/*L* as a function of the applied contact pressure $\sigma _{0}/E^{*}$ for different values of the retracting speed $\hat{V}$ and unloading starting from unrelaxed state; (**b**) The adimensional applied contact pressure $\sigma _{0}/E^{*}$ as a function of the normalized approach $\delta /h_{\textrm{rms}}$ for different values of the retracting speed $\hat{V}$ and unloading starting from unrelaxed state. Results are given for a self-affine fractal profile with Hurst exponent $H=0.8$, $h_{\textrm{rms}}=20$ nm, $\zeta =128$, and surface energy $\Delta \hat{\gamma }=0.025$.

Figure 2 depicts the viscoelastic pull-off stress $\sigma _{\mathrm {pull-off}}$, i.e., the maximum tensile stress reached during retraction, as a function of the speed $\hat{V}$. The pull-off is normalized with respect to its elastic value $\sigma _{\mathrm {pull-off,el}}$, obtained under quasi-static unloading conditions. It is observed that $\sigma _{\mathrm {pull-off}}$ increases monotonically with the retraction speed and eventually reaches a plateau at high $\hat{V}$. This finding is consistent with previous observations made for both smooth and single-scale rough indenters^7,13^. The upper bound value is restricted by the ratio $E_{\infty }/E_{0}$^8^, but it is also affected by size effects^39^, contact geometry and range of adhesion^40^. We also show the contact configurations at the pull-off instant, for four different unloading speeds ($\hat{V}=0,$ 0.333,  3.333,  333.33), corresponding to points A, B, C, D. In the case of smooth Hertzian line contacts, Müser and Persson^8^ found that the contact area at the pull-off instant decreases with increasing $\hat{V}$. The presence of roughness reverses this trend as we observe that the contact area increases with the speed at pull-off (see also Fig. 1a). Moreover, moving from configuration $\textrm{A}$ to D, we observe that increasing the speed the viscoelastic half-plane has no time to recover its initial shape, resulting in maintaining the shape of the indenter upon detachment. A similar behavior is observed in shape memory polymers (SMPs) with tunable elastic modulus and temporary shape locking. If the SMPs is deformed in the rubbery state and undergo a rubber-to-glass transition (R2G), they can temporarily lock in a deformed configuration (shape locking phenomenon). Furthermore, our results demonstrate a significant enhancement in the adhesion capability when the system is attached in the rubbery state and detached in the glassy state, which agrees with the experimental findings on SMPs presented in Ref.^41^. Therefore, in our case as well, we can identify a similar phenomenon to the shape locking effect, which is the main cause of the increased adhesion observed at high speeds.

**Figure 4:**
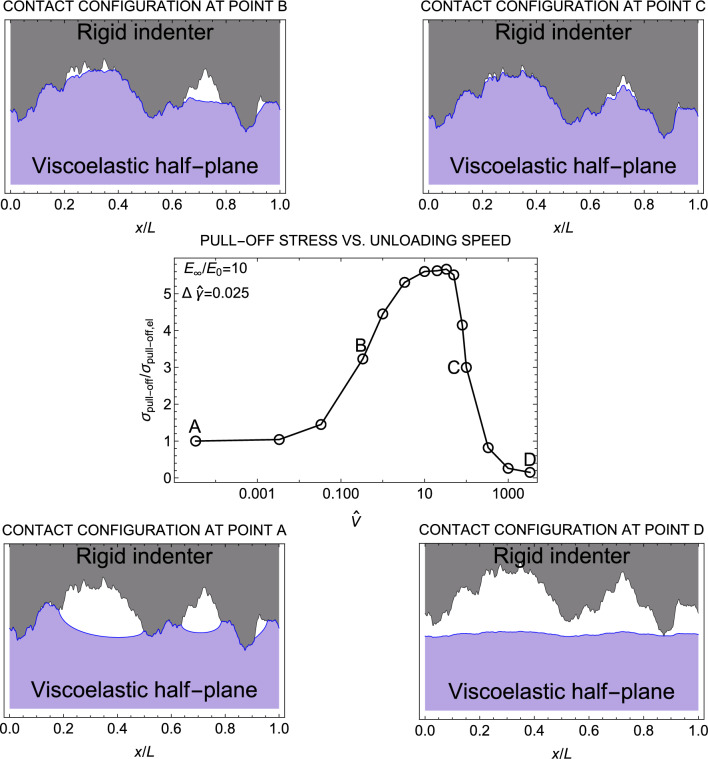
The pull-off stress $\sigma _{\mathrm {pull-off}}/\sigma _{\mathrm {pull-off,el}}$ normalized with respect to the elastic value as a function of the retracting speed $\hat{V}$ when unloading starts from unrelaxed state. Results are given in a semi-log plot and for a self-affine fractal profile with Hurst exponent $H=0.8$, $h_{\textrm{rms}}=20$ nm, $\zeta =128$, and surface energy $\Delta \hat{\gamma }=0.025$. In the figure, the deformed configuration of the half-plane at the pull-off instant is also shown for four unloading speeds ($\hat{V}=0,0.333,100,3333.33$).

A second set of simulations has been performed by fixing the same speed for the loading and unloading phases. In such case, unloading starts right after loading, from an unrelaxed state of the viscoelastic material. Figure 3a shows the normalized contact area *A*/*L* in terms of the adimensional pressure $\sigma _{0}/E^{*}$, while Fig. 3b shows the adimensional pressure $\sigma _{0}/E^{*}$ as a function of the normalized penetration $\delta /h_{\textrm{rms}}$. Similar to the retraction process, when the approach is performed at a non-zero speed, viscoelastic effects dampen the roughness-induced instabilities associated with jump-in phenomena. Furthermore, as the approach speed increases, the loading path exhibits reduced adhesion due to a decrease in effective surface energy at higher $\hat{V}$^42^. Consequently, the maximum hysteretic loss is not observed at the highest speeds $\hat{V}$, but at intermediate values.

Figure 4 shows the normalized pull-off stress in terms of the driving speed $\hat{V}$. The trend of the pull-off stress with $\hat{V}$ is the same of the adhesion hysteresis, and the maximum pull-off stress is found at an intermediate speed, consistent with previous findings by Afferrante and Violano^7^ for Hertzian contact. However, when a smooth flat punch indenter in considered, the dependence on the specific loading process details, such as maximum indentation at preload and loading rate, is lost as highlighted in Ref.^43^.

Figure 4 presents the deformed configurations corresponding to the pull-off instant for four unloading speeds ($\hat{V}=0,$ 0.333,  100,  3333.33). As we move from configuration $\textrm{A}$ to $\textrm{D}$, the debonding process no longer follows crack propagation but instead exhibits quasi-uniform bond breaking. This transition occurs due to the stiffer material response at higher $\hat{V}$, potentially leading to a shift from short-range to long-range adhesion and consequent uniform bond rupture. In line with observations made by Violano and Afferrante^40^ regarding smooth Hertzian contacts, short-range adhesion primarily involves localized viscous dissipation at the contact edge, resembling crack opening mechanisms. Conversely, in the long-range adhesion regime, dissipation occurs throughout the bulk material, resulting in a uniform debonding process. A similar phenomenon was also observed in Ref.^44^ for a linear viscoelastic material sliding at a constant velocity against a rough substrate. The pull-off force is predicted to be velocity-dependent, with a maximum value occurring at an intermediate sliding velocity, as the majority of energy dissipation takes place within the bulk of the material. Furthermore, as contacts transition towards the nano-scale, a shift from fracture-dominated to interfacial-strength-dominated pull-off behavior is observed^39,45^. In this context, Baker et al.^45^ showed that the pull-off force exhibits a dependence on the preload, as “nanoscale adhesion is governed by the product of adhesive strength and contact area”.

At higher speeds, the pull-off stress is reduced compared to its quasi-static value. When the loading-unloading speed is fast enough for the mechanical response of the substrate to fall into the glassy region, the material exhibits elastic behavior with an elastic modulus of $E_{\infty }$. In such cases, the substrate becomes less compliant, requiring a higher pressure to achieve full-contact conditions. Consequently, an increase in the elastic modulus is expected to result in a lower pull-off force. This finding may appear counter-intuitive since, in smooth Hertzian contacts, the pull-off force is typically considered independent of the elastic modulus^46^.

**Figure 5:**
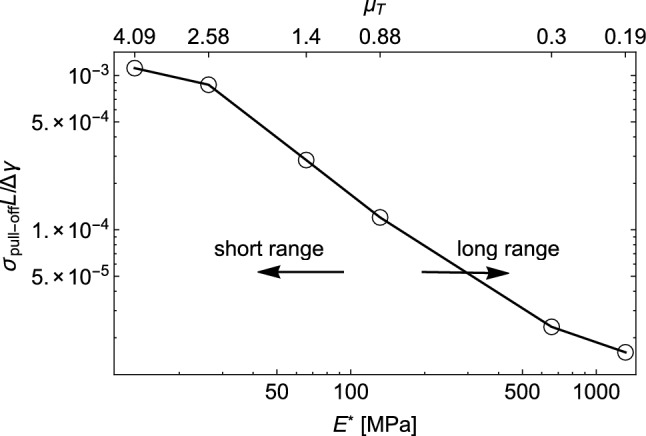
The adimensional pull-off stress $\sigma _{\mathrm {pull-off}} L/\Delta \gamma$ as a function of the reduced elastic modulus $E^{*}$ in the limit of elastic half-plane ($V\approx 0$). The values of the generalized Tabor parameter $\mu _{\text {T}}=R^{1/3}(\Delta \gamma /E^{*})^{2/3}/\epsilon$, are reported on the top axis. Results are given in a double logarithmic plot and for a self-affine fractal profile with Hurst exponent $H=0.8$, $h_{\textrm{rms} }=20$ nm, $\zeta =128$, and surface energy $\Delta \hat{ \gamma }=0.025$.

To further investigate this aspect, we performed loading-unloading simulations under quasi-static conditions ($\hat{V}\sim 0$), considering various values of the elastic modulus $E^{*}$. The results are collected in Fig. 5, where the pull-off stress is plotted as a function of the reduced elastic modulus $E^{*}$ on a double logarithmic scale. The top axis displays the values of the generalized Tabor parameter $\mu _{\text {T}}=R^{1/3}(\Delta \gamma /E^{*})^{2/3}/\epsilon$, being *R* the average radius of curvature of the rough profile. The pull-off stress exhibits a decrease as the elastic modulus $E^{*}$ increases. While this behavior is typically observed in smooth contacts during the transition from short- to long-range adhesion^47^, we find that it occurs in both regimes for rough contacts. In the case of rough contacts, this phenomenon is not directly related to the transition between short- and long-range adhesion, as observed in smooth contacts. Indeed, as the elastic modulus $E^{*}$ increases (i.e., the generalized Tabor parameter decreases), a greater number of roughness scales experience long-range adhesion locally. Consequently, the transition from the short- to long-range adhesion regime in rough contacts is smoother due to the presence of multiscale roughness, compared to smooth contacts. Interestingly, a similar trend of the pull-off force with the elastic modulus has been experimentally observed by Dalvi et al.^6^ in the short-range adhesion regime for PDMS-glass contacts.

**Figure 6:**
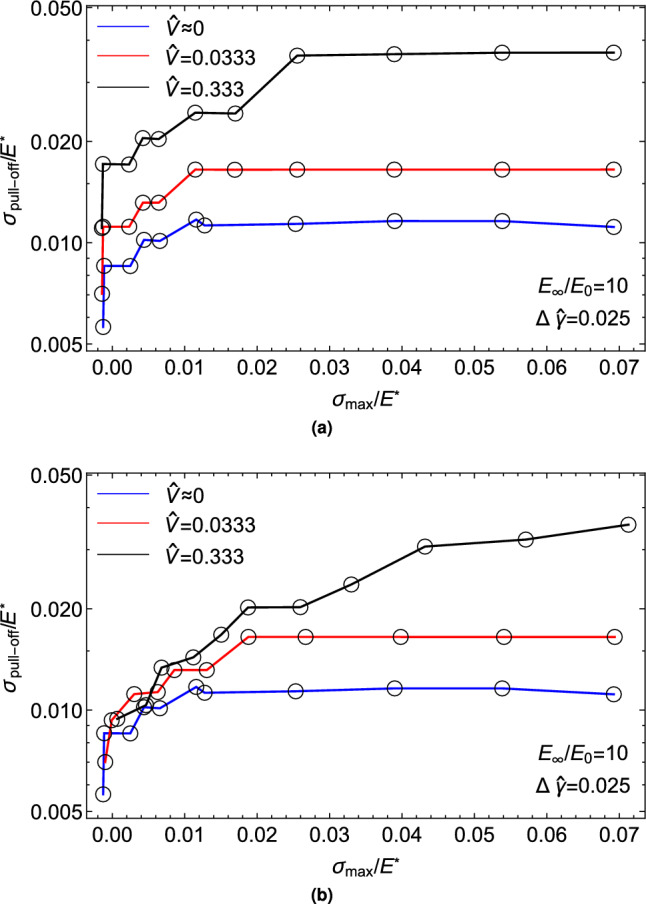
The pull-off stress $\sigma _{\mathrm {pull-off}}/E^{*}$ as a function of maximum load $\sigma _{max}/E^{*}$ for unloading from (**a**) relaxed and (**b**) unrelaxed state. Results are given in a semi-log plot and for a self-affine fractal profile with Hurst exponent $H=0.8$, $h_{\textrm{rms}}=20$ nm, $\zeta =128$, and surface energy $\Delta \hat{\gamma }=0.025$.

In the case of axisymmetric Hertzian contacts, Violano and Afferrante demonstrated that the viscoelastic pull-off force is influenced by a combination of rate and size effects^39^. When viscous effects are not negligible, they observed a dependence of the pull-off force on the maximum load. In this regard, Fig. 6 shows the pull-off stress as a function of the maximum load $\sigma _{max}/E^{*}$ for both unloading from a relaxed (Fig. 6a) and an unrelaxed (Fig. 6b) state. It is evident that the pull-off force increases monotonically with $\sigma _{max}/E^{*}$. Moreover, in contrast to the smooth case, the pull-off stress exhibits load dependency during quasi-static retraction ($\hat{V}\sim 0$), in agreement with the experimental findings of Dorogin et al.^48^. They noted that this effect is associated with “*the adhesion hysteresis that occurs in the condition of incomplete contacts induced by roughness*”. Finally, it is worth noting that once complete contact is achieved, further increasing $\sigma _{max}$ beyond the value of complete contact has no effect on the pull-off stress.

## Discussion

The detachment of a rigid rough indenter from a viscoelastic half-plane exhibits complex behavior that is influenced by the loading process.

When unloading starts from the relaxed state of the material, both adhesion hysteresis and pull-off stress increase monotonically with the retraction speed until reaching a plateau when the material response enters the glassy region. When viscous effects are negligible, i.e., when retraction is performed under quasi-static conditions, adhesion hysteresis is primarily caused by multiple instabilities related to jumps in and out of contact. Viscoelasticity dampens these effects but does not eliminate them, even at high retraction speeds.

When unloading starts from an unrelaxed state of the material, i.e., when approach and retraction are performed at the same non-zero velocity without dwell time, the pull-off stress and adhesion hysteresis exhibit a bell-shaped trend with the retraction speed. Additionally, viscoelasticity is observed to have the effect of suppressing roughness-induced instabilities even during the loading phase.

Contrary to expectations for smooth contacts, the pull-off stress decreases with an increase in the composite elastic modulus of the system. This can be attributed to stiffer systems requiring more elastic energy to achieve full-contact conditions. During retraction, this energy is released, aiding in the breaking of adhesive bonds and reducing the pull-off stress^18^. Finally, the pull-off stress is observed to increase with the maximum load due to the roughness-induced hysteresis that occurs in the condition of incomplete contact^48^. This effect is significantly amplified by the presence of viscoelasticity.

Our results demonstrate that interface adhesion can be controlled by modulating the excitation frequency, which affects both adhesion hysteresis and dynamic instability. These findings are consistent with the experimental observations presented in Ref.^49^, where continuous regulation of interface adhesion was achieved by inducing mechanical micro-vibrations in the adhesive system.

## Methods

**Figure 7:**
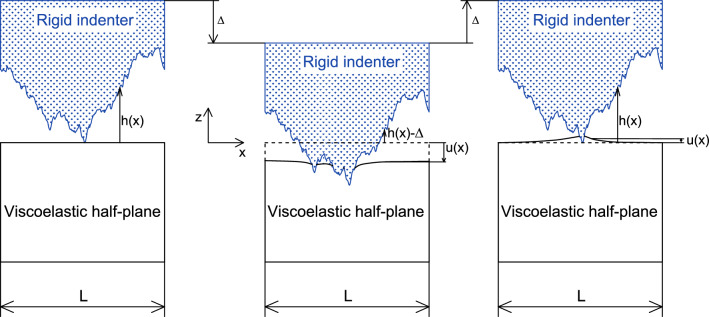
The problem under investigation: a rigid randomly rough 1D profile is pressed into a linear viscoelastic half-plane and then pulled apart from it.

Figure 7 shows a sketch of the problem under investigation: a rigid randomly rough 1D profile is pressed into a linear viscoelastic half-plane and then pulled apart from it. The rough profile is assumed to be periodic with period *L* and the quantities *h* and *u* are, respectively, the heights distribution of the rough profile and the interfacial normal displacement of the viscoelastic half-plane occurring when it is squeezed of $\delta$ by the rigid indenter.

### Generation of the rough profiles

Spectral methods^50,51^ are employed for the numerical generation of the rough profile, which is described by a self-affine fractal geometry with a power spectral density (PSD) given by

1 $$\begin{array}{*{20}l} {C\left( q \right) = {\mkern 1mu} C_{0} } \hfill & {{\text{for }}q_{{\text{L}}} \le q < q_{{\text{r}}} } \hfill \\ {C\left( q \right) = C_{0} \left( {q/q_{{\text{r}}} } \right)^{{ - \left( {1 + 2H} \right)}} } \hfill & {{\text{for }}q_{{\text{r}}} \le q < q_{{\text{s}}} } \hfill \\ \end{array}$$

and zero otherwise. The quantity *q* is the wavenumber and $q_{\textrm{L} }=2\pi /L$ and $q_{\textrm{s}}=2\pi /\lambda _{\textrm{s}}$ are, respectively, the short and long frequencies cut-off, while $q_{\textrm{r} }=2\pi /\lambda _{\textrm{r}}$ is the roll-off frequency. The parameter *H* is the Hurst exponent, which is related to the fractal dimension $D=2-H$.

The rough profile is then described by the Fourier series

2 $$\begin{aligned} h\left( x\right) =\sum _{k=1}^{N}h_{\textrm{k}}\cos \left( kq_{\textrm{L} }x+\varphi _{\textrm{k}}\right) , \end{aligned}$$

where the random phases $\varphi _{\textrm{k}}$ are uniformly distributed on the interval $[-\pi$, $\pi ]$ to satisfy the translational invariance of the profile statistical properties.

### Finite element model

The contact mechanics problem is addressed using the finite element model developed in Ref.^7^, with the aid of the ANSYS APDL software.

The rough rigid indenter is defined by specifying the nodes on its profile. It utilizes a single master node to apply force and displacement, with its degrees of freedom linked to those of all other nodes through constraint equations.

Adhesive interactions are modeled exploiting the so-called Derjaguin approximation^52^ and using nonlinear springs with a traction-displacement relation based on the Lennard-Jones potential law,

3 $$\begin{aligned} \sigma \left( x\right) =\frac{8\Delta \gamma }{3\varepsilon }\left[ \left( \frac{\varepsilon }{g\left( x\right) }\right) ^{3}-\left( \frac{\varepsilon }{ g\left( x\right) }\right) ^{9}\right] \end{aligned}$$

where $g\left( x\right)$ is the interfacial gap, $\Delta \gamma$ the surface energy of adhesion, and $\varepsilon$ the range of action of the van der Waals forces. A similar methodology has been utilized in Ref.^53^, where the 9-3 Lennard-Jones potential was employed to describe interactions between atoms. Loading-unloading cycles are simulated by controlling the driving speed V of the master node in the rigid profile. As a result, the total contact force is determined as the reaction force to the applied displacement. We verified that this value corresponds to the sum of contributions arising from the deformation of each spring. This approach was first proposed by Muller et al.^54^, who utilized numerical summation of interactions to compute the force acting between a smooth sphere and a flat surface.

The viscoelastic half-plane is modeled with linear isoparametric plane strain elements. Periodic boundary conditions are applied to the lateral edges of the system, and the viscoelastic substrate is fully constrained at the bottom. The height of the substrate is assumed to be larger than 10 *L*, in order to approximate the behavior of a half-plane. We have verified that the results remain consistent even with a further increase in this value. A standard linear solid is used to describe viscoelasticity in the half-plane, according to Maxwell representation

4 $$\begin{aligned} E\left( t\right) =E_{0}+\left( E_{\infty }-E_{0}\right) \exp \left( -t/\tau \right) \end{aligned}$$

where $E_{0}$ and $E_{\infty }$ are Young’s moduli at zero and high frequency, respectively. Note $\tau$ is the relaxation time and is related to the creep time $\tau _{\textrm{c}}$ by $\tau =\tau _{\textrm{c} }E_{0}/E_{\infty }$. The stress $\mathbf {\sigma }$ is calculated according to the constitutive equation

5 $$\begin{aligned} \mathbf {\sigma =}\int _{0}^{t}E\left( t-t^{\prime }\right) \frac{d\mathbf { \epsilon }}{dt^{\prime }}dt^{\prime } \end{aligned}$$

where $\mathbf {\epsilon }$ is the strain and $E\left( t\right)$ is the relaxation function.

During the approach of the indenter, multiple snap-through events occur as a result of attractive forces. These events can lead to numerical convergence issues, particularly at slow driving speeds of the indenter, where viscoelasticity does not dampen contact instabilities. To ensure numerical stability, we approach the quasi-static problem as a “slow dynamic” analysis. In this approach, we incorporate dashpot and mass elements at the interface, using point masses of $10^{-5}$ kg and dashpots with a damping constant of $10^{12}$ kg/s. Additionally, appropriate time-integration parameters are carefully selected to prevent divergence (a time step increment of $\Delta t=0.1$ s is used, except in cases of contact instability where the time step is reduced by up to 1000 times). The damping and inertia forces do not alter the contact solution as they are activated solely during the snap-through events.

### Topography and material properties

In this study, we use rigid self-affine profiles characterized by a wavelength cut-off $\lambda _{\textrm{L}}=L= 4\times 10^{-6}$ $\textrm{m}$ and roll-off $\lambda _{\textrm{r}}=0.5 L$. The presence of a roll-off region is common in most manufactured and natural rough surfaces, where the Power Spectral Density (PSD) value remains nearly constant^55^.To represent real surfaces, we adopt a Hurst exponent $H = 0.8$, which is a commonly observed value^55^. The RMS roughness amplitude is set to the nanometric value $h_{\textrm{rms}}=20$ nm, which is typical for manufactured surfaces subjected to modern machine tooling^56^ and biomedical implants^57^. Furthermore, we fix a magnification factor of $\zeta =\lambda _{\textrm{L}}/\lambda _{\textrm{s}}=128$ is fixed, with $\lambda _{\textrm{s}}$ represents the smallest wavelength in the roughness spectrum.

The substrate is modeled with a nearly incompressible viscoelastic material ($\nu =0.49$), as expected for real rubbers and silicones^3^. The ratio of the Young’s moduli at zero and high frequencies is fixed at $E_{0}/E_{\mathrm {\infty }}=0.1$, and the single relaxation time is set to $\tau =10^{-4}$ s. Although real viscoelastic materials may exhibit lower values of $E_{0}/E_{\mathrm {\infty }}$ and more relaxation times^58^, the qualitative rheological behavior remains consistent. The range of action of van der Waals forces is specified as $\epsilon =3$ nm. Such a value is chosen based on previous experimental studies^59–61^, where $\epsilon$ has been found to range between 0.3-5 nm. Finally, we introduce an interfacial surface energy $\Delta \gamma = 0.05$ J/m$^2$, which is close to the value measured for a PDMS-glass interface^4^.

## Data Availability

The datasets used and/or analyzed during the current study available from the corresponding author on reasonable request.
